# Peer-led training improves lifejacket wear among occupational boaters: Evidence from a cluster randomized controlled trial on Lake Albert, Uganda

**DOI:** 10.1371/journal.pone.0292754

**Published:** 2023-10-20

**Authors:** Frederick Oporia, Simon P. S. Kibira, Jagnoor Jagnoor, Olive Kobusingye, Fredrick Edward Makumbi, John Bosco Isunju, Fred Nuwaha

**Affiliations:** 1 Department of Disease Control and Environmental Health, Makerere University School of Public Health, Kampala, Uganda; 2 Department of Community Health and Behavioural Sciences, Makerere University School of Public Health, Kampala, Uganda; 3 The George Institute for Global Health, University of New South Wales, Camperdown, New South Wales, Australia; 4 Department of Epidemiology and Biostatistics, Makerere University School of Public Health, Kampala, Uganda; United States Environmental Protection Agency, UNITED STATES

## Abstract

**Background:**

The burden of drowning among occupational boaters in low and middle-income countries is highest globally. In Uganda, over 95% of people who drowned from boating-related activities were not wearing lifejackets at the time of the incident. We implemented and evaluated a peer-led training program to improve lifejacket wear among occupational boaters on Lake Albert, Uganda.

**Methods:**

We conducted a two-arm cluster randomized controlled trial in which fourteen landing sites were randomized to the intervention and non-intervention arm with a 1:1 allocation ratio. In the intervention arm, a six-month peer-to-peer training program on lifejacket wear was implemented while the non-intervention arm continued to receive the routine Marine Police sensitizations on drowning prevention through its community policing program. The effect of the intervention was assessed on self-reported and observed lifejacket wear using a test of differences in proportions of wear following the intention to treat principle. The effect of contamination was assessed using mixed effect modified Poisson regression following the As Treated analysis principle at 95% CI. Results are reported according to the CONSORT statement–extension for cluster randomized trials.

**Results:**

Self-reported lifejacket wear increased markedly from 30.8% to 65.1% in the intervention arm compared to the non-intervention arm which rose from 29.9% to 43.2%. Observed wear increased from 1.0% to 26.8% in the intervention arm and from 0.6% to 8.8% in the non-intervention arm. The test of differences in proportions of self-reported lifejacket wear (65.1%– 43.2% = 21.9%, p-value <0.001) and observed wear (26.8%– 8.8% = 18%, p-value <0.001) showed statistically significant differences between the intervention and non-intervention arm. Self-reported lifejacket wear was higher among boaters who received peer training than those who did not (Adj. PR 1.78, 95% CI 1.38–2.30).

**Conclusion:**

This study demonstrated that peer-led training significantly improves lifejacket wear among occupational boaters. The government of Uganda through the relevant ministries, and the Landing Site Management Committees should embrace and scale up peer-led training programs on lifejacket wear to reduce drowning deaths.

## Background

Drowning has plateaued as the third leading cause of unintentional injury deaths globally, accounting for over 7% of all injury fatalities [1, 2]. Over the past decade, there has been a gradual reduction in the global number of annual drowning deaths from 372,000 in 2012 [1] to the current estimate of 236,000 [2]. This burden continues to be disproportionately distributed in low and middle-income countries (LMICs) where over 90% of the deaths occur [1]. These global estimates exclude drownings from water transport and water-related disasters which have become more pronounced in the nexus of climate change [3]. The toll is especially greatest among people involved in occupational boating activities such as fishing and water transport. The human cost of fishing exceeds 100,000 annual deaths among fisherfolk, the majority of which occur in the World Health Organization (WHO) African region, especially Sub-Saharan Africa [4]. Indeed, available data shows that Uganda suffers the world’s highest annual drowning death rate recorded in lakeside fishing and boat using communities estimated at 502 per 100,000 population [5, 6]. Drowning therefore in this context is an occupational problem as opposed to a leisure hazard in high-income countries (HICs).

Upon immersion in water, multiple physiological responses are evoked. The first is a cold shock that may lead to hyperventilation, muscle spasms, increased heart rate, loss of respiratory control, and cardiac arrest. This is followed by swimming failure, hypothermia, and finally post-rescue collapse [7, 8]. Without immediate rescue and proper resuscitation, heart failure can occur within two minutes of total immersion and onset of respiratory impairment [9]. While lifejackets are over 80% effective in preventing drowning [10, 11], their greatest efficacy is only realized before hypothermia sets in [7]. In fact, regardless of one’s swimming ability and water temperature, survival time in water when wearing a lifejacket is over 7 times longer and can range from 5–36 hours when the water temperature is 15°C, and even much longer when the water is at room temperature of about 20°C to 22°C [7]. Despite this high efficacy, lifejacket wear is low in both HICs and LMICs [6, 12–14]. Over 95% of the people who drowned from a boating-related activity in Uganda were not wearing one at the time of the incident [15]. This was attributed to inadequate knowledge about the importance and benefits of lifejackets among lakeside boat-using communities [5, 15–17].

The optimal performance of a lifejacket is affected by behavioral factors including incorrect or inconsistent use [1, 6], which are largely driven by insufficient knowledge about its benefits and donning procedures [5, 15, 16]. A lifejacket must be correctly donned and well-fitting to achieve its optimal performance [7, 10, 11]. An extensive review of the literature shows that interventions on drowning prevention have been focused on leisure and recreational boaters mostly in HICs [18–20], with hardly any targeting occupational drowning prevention in rural low-resource settings. Interventions on improving lifejacket wear among occupational boaters are extremely rare in both high-income countries (HICs) and LMICs [10, 20, 21]. Yet, the few that have been conducted have focused on fishers in HICs [22, 23], and cannot be generalized to low-income communities. In addition, Uganda is still deficient in policies and regulations to enforce the new law on maritime safety [24]. This study aimed to implement and evaluate a peer-led training intervention program to improve lifejacket wear to prevent drowning among boaters involved in occupational boating activities on Lake Albert in western Uganda.

## Methods

### Study design

We implemented a two-arm cluster randomized controlled trial [25, 26] for two reasons: (I) the individuals in the landing sites mix and interact with others and (II) the intervention was to be administered at group level. A cluster included all boaters residing in the landing site at the time of the study.

### Randomization

**Fig 1:**
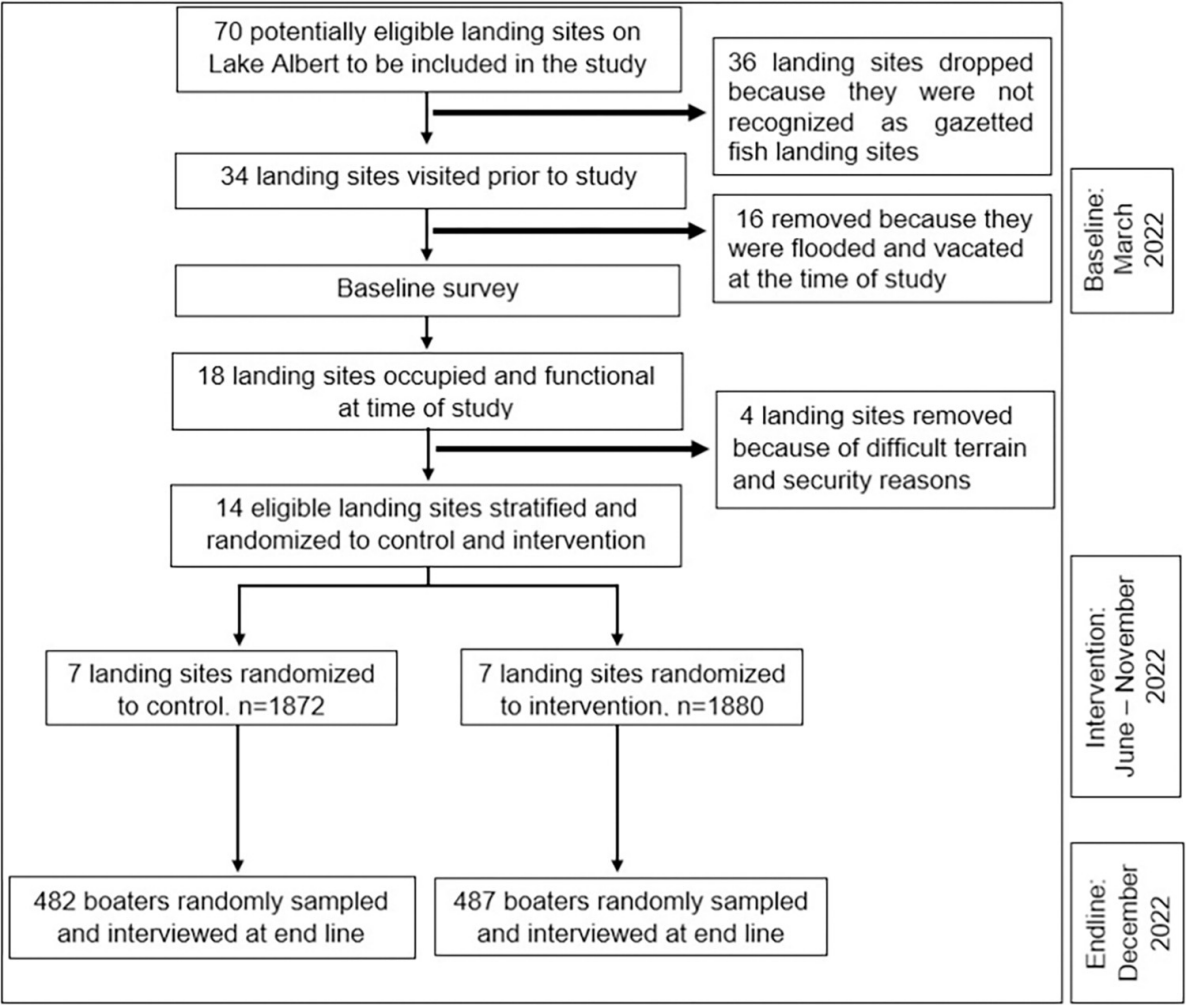
Randomization schema for the cluster randomized controlled trial on Lake Albert, Uganda.

Stratified permuted block randomization [25, 27, 28] was conducted, with landing sites as the randomization unit using a 1:1 allocation ratio. The randomization schema is illustrated on **Fig 1.** The recommended minimum number of clusters per arm is seven [28]. The cluster design was chosen to avoid contamination at individual-level randomization because the intervention was not blinded. The landing sites were stratified by estimated boater population size and baseline prevalence of lifejacket wear [29]. These stratification variables were chosen because this was not an individual follow-up study, meaning that a person wearing a lifejacket at baseline may not necessarily wear it at endline (after six months). It was also not guaranteed that a person interviewed at baseline would be the same person interviewed at endline because of the random sampling as well as a possible effect of regression to the mean (RTM) [30].

Stratification was aimed at increasing baseline comparability [25, 26]. We compared the prevalence of baseline self-reported lifejacket wear between the two arms using a test of differences in proportions. A p<0.05 indicated a significant difference. A geographical buffer was used to reduce the risk of contamination by leaving at least one landing site which was not part of the study in between an intervention and non-intervention site. From each stratum, one cluster was randomized to the intervention arm and the other to the non-intervention arm. The randomization was done using block sizes of 2, 4, and 8 by an independent statistician who was not part of the study team. The multiple blocks were used to improve randomness (reduce predictability) [31, 32].

#### Study setting and population

**Fig 2:**
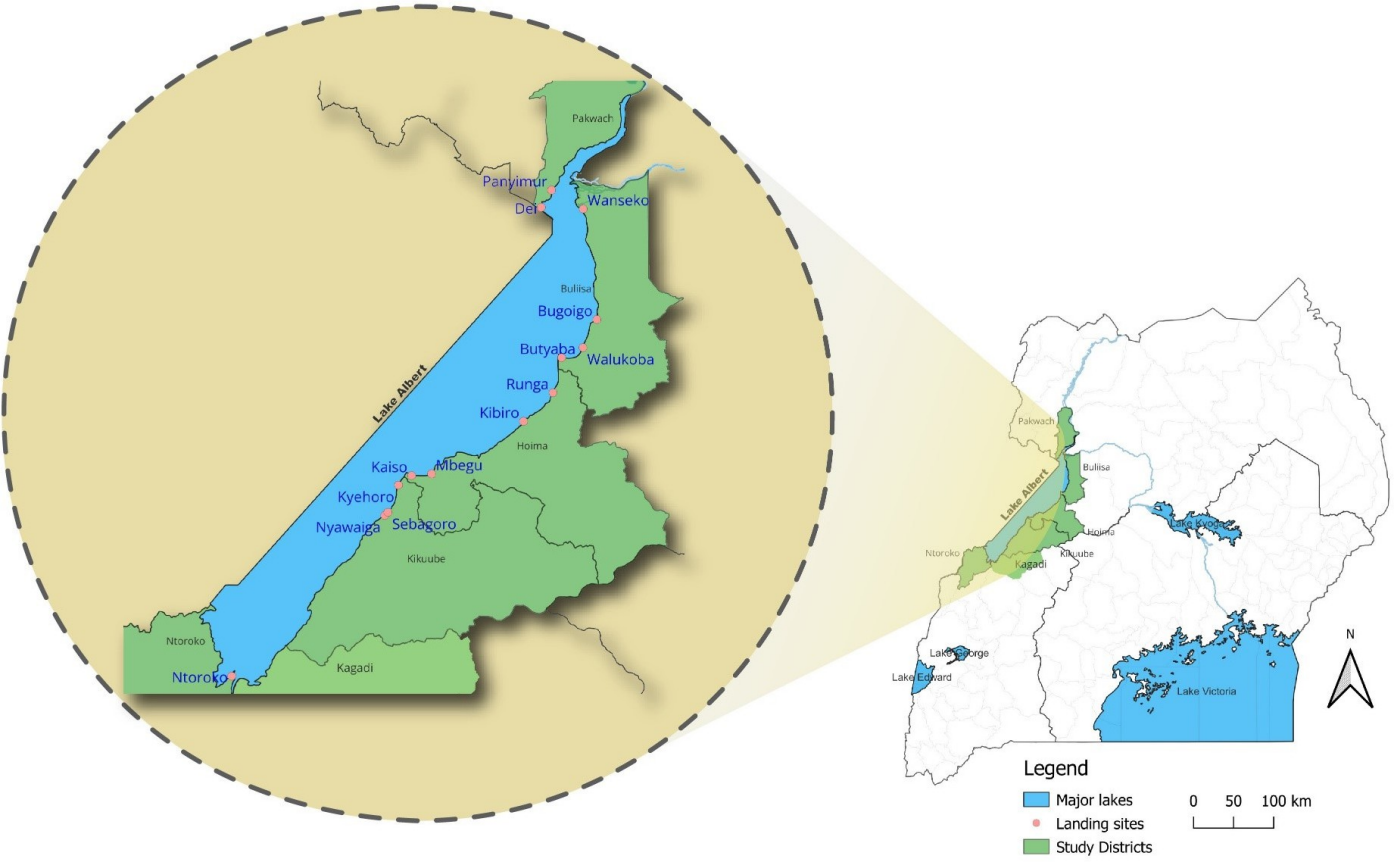
Map illustrating the location of the study sites.

This study was conducted among boaters involved in occupational boating activities on Lake Albert in western Uganda. These were fishermen and operators of transport boats, in this study collectively referred to as occupational boaters. Lake Albert is Africa’s seventh-largest freshwater body, located at the border between Uganda and the Democratic Republic of Congo [33]. The lake supports the local livelihoods of about four million people on the Ugandan side who mainly depend on fishing and water transport services [34]. The landing sites are at the shorelines of the lake and are used by boaters for embarking and disembarking (docking), and are spread across five districts: Kikuube, Hoima, Ntoroko, Buliisa, and Pakwach. The sites are also business hubs as well as clusters of residences for the locals. Lifejackets and other personal floatation devices (PFDs) are among the merchandize sold at the landing sites. According to the National Fisheries Resources Research Institute and Uganda Police Marines, there are over 70 landing sites on the Ugandan side but only about half are gazetted and recognized as fishing landing sites. However, due to the rising lake water levels, many of the gazetted sites had been flooded and vacated, leaving only 18 accessible and occupied at the time of this study, out of which only 14 were eligible for randomization **(Fig 2).** The four were left out due to security concerns and difficult geographical terrain that made them hard to access. We preferred the gazetted landing sites because they have established leadership structures that made it easier and safer for the study team. Other details are described in the baseline studies [16, 29].

#### Sample size determination

All the 14 landing sites that were eligible at the time of this study were included (Fig 2). Therefore, the number of clusters was not determined using any formula. To compute the number of individuals interviewed per arm, the author used the sample size formula for comparing two proportions [35] with the following assumptions: a two-sided Z_α/2_ of 0.05 for a 95% confidence interval, and Z_β_ statistical power of 90%. The proportion of individuals wearing lifejackets was obtained from the baseline survey at 32% [29], with the hope of raising it to 55% at endline. These assumptions yielded a total of 97 boaters per arm. This was inflated by a design effect of 5.1 calculated using the intra-cluster correlation coefficient (ICC) obtained from the baseline survey [29] and an average cluster size of 35. These assumptions yielded a final sample size of 495 boaters per arm, giving an overall total of 990 boaters across 14 clusters.

#### Study hypothesis

We hypothesized that the difference in the effectiveness of a peer-led training intervention program and the routine marine community policing program on lifejacket wear was zero.

#### Sampling procedures

The respondents were sampled using proportionate to boater-population size sampling techniques to obtain the required number of boaters per cluster. Study participants were identified from their shelters at the landing sites. The sampling frame was obtained from the landing site management committee. At each shelter, simple random sampling was used to select the boaters for interview. At each landing site, the interviews started at about 7:30 am (the time many boaters return from the lake) to 4:30 pm (the time they start to go back to the lake). Diligence was taken to ensure that no participant (boater) was interviewed more than once.

#### The peer-led training intervention

The peer-led, also referred to as peer-to-peer training intervention program was identified through a participatory approach in a stakeholder insight workshop. At least ten peers from each of the intervention sites were selected and trained for two days to become trainers of their colleagues through formation of ‘lifejacket clubs’. A peer was defined as a boater who is a drowning survivor who was wearing a lifejacket at the time of the incident, and this should have occurred in the last two years. The two years were preferred to minimize recall bias as they shared their experiences with their colleagues during the lifejacket wear promotion sessions. In situations where a drowning survivor was not available, the replacement was a person who was a consistent user of a lifejacket. A consistent user was defined as a person who reported having worn a lifejacket for all the last five boat trips to the lake, and with proof of having lifejacket. In addition, the peers had to be known members of the landing site, with no known criminal record. These were chosen so that they could share their experiences about drowning and lifejacket wear and to improve their credibility. All the peers were volunteers who acted in the spirit of citizen watch for safety on the lake.

The peers were trained to train their colleagues on various aspects of lifejackets and lifejacket wear. The training largely focused on observation of pre-departure safety precautions, most important of which was having a lifejacket on by all the boat occupants. They trained their colleagues on the importance of wearing lifejackets correctly, emphasized by regular reminders. Correct lifejacket donning procedures were taught through demonstrations. Emphasis was put on wearing a lifejacket, regardless of the seaworthiness of the lifejacket. The training was conducted continuously for six months by the resident peers at their respective landing sites. General sessions were held every two weeks at each of the intervention sites. This frequency was chosen with the aim of ensuring ‘adequacy of the intervention’ to provide a detectable dose-response relationship. In addition, the fortnightly training sessions were hoped to minimize message fatigue so people do not get ‘irritated’ by the same messages. This frequency was successful in a previous quasi experimental study among similar fishing communities on Lake Victoria in central Uganda [36]. All the peer trainers were provided with notebooks and pens to record the details of the trainings they have conducted (e.g., date of training, number of people trained, topic taught, challenges etc.) Both the intervention and non-intervention arm continued receiving the routine Uganda Police messages on water safety through its community policing program. In its routine community policing program, the Uganda Police Force (UPF) conducts sensitizations at the landing sites on water safety including lifejacket use.

#### Data collection and measurement

We did a repeat survey after six months using the same tools and approach described in detail in the baseline study [29]. We collected data using an electronic questionnaire uploaded on open data kit (ODK) software installed on android tablets with appropriate restrictions and branching logic to avoid missing variables. The questionnaire was pre-tested at Ggaba landing site on Lake Victoria and minor revisions were made. The purpose of pre-testing was for clarity, suitability, validation and logical flow of the items. No major revisions were made to the questionnaire. The primary outcome was lifejacket wear which was assessed through individual interviews with the boaters to obtain self-reported wear as well as observed wear obtained through covert (discreet) observations as the boaters disembarked from the lake. The discreet observations were preferred to avoid the risks of the hawthorne effect [37]. Boaters who were observed wearing lifejackets as they disembarked from their boats on the lake were entered into ODK observation checklist using android tablets. The observations and interviews were conducted concurrently by different teams of data collectors.

Self-reported lifejacket wear was measured as a binary variable: boaters who “always wore a lifejacket while on the lake” were considered to have self-reported lifejacket wear, and were assigned to “yes” category and the rest to “no” category. The secondary outcome was self-reported lifejacket ownership which was assessed through interviews with the boaters. Because the sampling was random, the boater who were observed were not necessarily same who were interviewed for self-reported wear. Four Research Assistants (RAs) were trained about all aspects of data collection, objectives, protocol, and standard operating procedures. The RAs were provided with a pictorial manual of the different types of lifejackets and boats for reference while in the field. They had a minimum of bachelor’s degree, and were native speakers of the languages most spoken in the study area. Single blinding was done to maintain internal validity and reduce exposure identification and interviewer bias by using RAs who did not know which clusters were in the non-intervention or intervention arm.

#### Data management and analysis

The data were imported into STATA15 software for further cleaning and analysis. Basic analysis followed the intention to treat (ITT) principle [38] to compare the prevalence of lifejacket wear in the intervention arm and that of the non-intervention arm. This was done through a test of differences in proportions of lifejacket wear following a linear regression principle and p-value of <0.05 was used to signify statistically significant differences. The ITT population included all the boaters who were interviewed in the intervention arm and all those interviewed in the non-intervention arm. To control the influence of contamination, further analysis was performed where the prevalence of self-reported lifejacket wear was adjusted for exposure to intervention following the As Treated (AT) analysis principle [39]. The AT population included all boaters who received the peer training on lifejacket wear and those who did not receive, regardless of the study arm they were as per this sample.

A multilevel mixed effect modified Poisson regression with level one as boaters and level two as cluster (landing sites) was employed to assess the effect of the intervention on lifejacket wear. Random landing site intercepts assuming a normal distribution with an unstructured covariance structure combined with fixed effects covariates were employed. Before performing the multilevel analyses, we ran a null model to calculate the intra-cluster correlation coefficient (ICC), which reflects the proportion of total variance in lifejacket wear outcome explained by the landing site. An ICC value of 0.082 was obtained, suggesting that a considerable amount of variation was accounted for by the landing site [40–43]. This justified the use of the mixed effects analysis [44–47] using a modified poisson regression model. Based on literature, an ICC value of below 0.5 indicates poor reliability while that above 0.75 indicates good reliability [42]. These results show a much lower ICC, meaning that there is high variability between the clusters.

We employed the logical model-building procedure using the backward elimination method in the multiple regression analysis. Variables that met the 0.2 level at bivariable analysis, as well as those in the conceptual framework, guided the model building procedure. The goodness of fit (GOF) of the model was assessed using the Hosmer-Lemeshow (HL) test. A level of 5% with a two-tailed test was used to signify statistical significance at 95% confidence interval. The reporting of the results was guided by the consolidated standards of reporting randomized-controlled trials (CONSORT) statement–extension for cluster randomized trials [48].

### Ethical considerations

This study was approved by the Research and Ethics Committee of Makerere University School of Public Health and registered with the Uganda National Council for Science and Technology (UNCST), registration #SS992ES. The study protocol was also registered with ClinicalTrials.gov, ID number NCT05337761. Administrative clearance from the leadership of the landing sites was obtained before the study. Only adults (≥18 years) and emancipated minors were recruited through a written informed consenting process. All data were kept confidential and only accessed by the study team.

## Results

### Socio-demographic characteristics of occupational boaters on Lake Albert, Uganda

A response rate of 98% (969/990) was obtained in the endline survey. The majority of the respondents sampled were aged 20–29 years, at baseline, non-intervention (36.1%) vs intervention (41.0%), and at endline, non-intervention (42.1%) vs intervention (42.7%). The largest proportion of the respondents in both arms were fishermen, and many were living with a spouse. In general, most of the respondents had attended formal education while a sizeable proportion was not trained to operate a boat, Table 1.

**Table 1:** Socio-demographic characteristics of occupational boaters on Lake Albert, Uganda.

Variable		Baseline				Endline			
		Non-intervention, N = 518 (%)		Intervention, N = 629 (%)		Non-intervention, N = 482 (%)		Intervention, N = 487 (%)	
Age (years)	Median age (IQR)	32	(26–41)	31	(26–39)	30	(25–38)	32	(26–39)
	Less than 20	8	(1.5)	18	(2.9)	21	(4.4)	4	(0.8)
	20 to 29	187	(36.1)	258	(41.0)	203	(42.1)	208	(42.7)
	30 to 39	178	(34.4)	201	(32.0)	151	(31.3)	162	(33.3)
	40 to 49	91	(17.6)	93	(14.8)	71	(14.7)	71	(14.6)
	50 and above	54	(10.4)	59	(9.4)	36	(7.5)	42	(8.6)
Education level	None	136	(26.3)	145	(23.1)	22	(4.6)	16	(3.3)
	Primary	21	(4.1)	18	(2.9)	353	(73.2)	360	(73.9)
	Secondary & above	361	(69.7)	466	(74.1)	107	(22.2)	111	(22.8)
Marital status	Single	98	(18.9)	144	(22.9)	115	(23.9)	99	(20.3)
	Living with spouse	420	(81.1)	485	(77.1)	367	(76.1)	388	(79.7)
Occupation	Fisherman	464	(89.6)	563	(89.5)	466	(96.7)	468	(96.1)
	Transporter	54	(10.4)	66	(10.5)	16	(3.3)	19	(3.9)
Occupancy of dwelling unit	Owner	252	(48.6)	256	(40.7)	177	(36.7)	176	(36.1)
	Renting	266	(51.4)	373	(59.3)	305	(63.3)	311	(63.9)
Frequency on the lake	Daily	241	(46.5)	300	(47.7)	307	(63.7)	302	(62.0)
	Few days in a week	230	(44.4)	292	(46.4)	139	(28.8)	157	(32.2)
	Once a week	47	(9.1)	34	(5.4)	36	(7.5)	28	(5.7)
Trained to operate a boat	Not trained	45	(8.7)	88	(14.0)	113	(23.4)	145	(29.7)
	Trained by a friend	473	(91.3)	541	(86.0)	369	(76.6)	342	(70.3)

### Lifejacket wear and ownership

Self-reported lifejacket wear was reported for 969 boaters who were interviewed, while the covert observation was done on 964 boaters across 14 landing sites as they disembarked from the lake in their boats. After a six-month peer-led training program, self-reported lifejacket wear rose from 30.8% at baseline to 65.1% (95% CI 60.7%– 69.2%) in the intervention arm, and from 29.9% to 43.2% (95% CI 38.8%– 47.6%) in the non-intervention arm. Observed lifejacket wear rose from 1.0% at baseline to 26.8% (95% CI 22.7%– 30.9%) at endline in the intervention arm, and from 0.6% to 8.8% (95% CI 6.5%– 11.6%) in the non-intervention arm. More than a half, 53.5% (95% CI 49.1%– 57.9%) and nearly three quarters, 71.5% (95% CI 67.3%– 75.3%) of the boaters reported having a lifejacket in the non-intervention and intervention arm respectively. The majority 81.5% (95% CI 77.8%– 84.7%) of the boaters in the intervention arm intended to wear a lifejacket on their next trip to the lake. Table 2.

**Table 2:** Prevalence of lifejacket wear and ownership among occupational boaters at endline.

Self-reported lifejacket wear	Non-intervention, N = 482		Intervention, N = 487	
	Yes	(95% CI)	Yes	(95% CI)
Have a lifejacket	53.5%	(49.1%– 57.9%)	71.5%	(67.3%– 75.3%)
Report wearing lifejacket	43.2%	(38.8%– 47.6%)	65.1%	(60.7%– 69.2%)
Wore a lifejacket on the last boat trip	51.9%	(47.4%– 56.3%)	73.1%	(68.9%– 76.9%)
Intend to wear lifejacket on next boat trip	64.1%	(59.7%– 68.3%)	81.5%	(77.8%– 84.7%)
**Observed lifejacket wear**	**Non-intervention, N = 490**		**Intervention, N = 474**	
	**Yes**	**(95% CI)**	**Yes**	**(95% CI)**
Observed wearing a lifejacket on boat	8.8%	(6.5%– 11.6%)	26.8%	(22.9%– 30.9%)

### Effect of peer-led training on lifejacket wear among occupational boaters on Lake Albert

**Fig 3:**
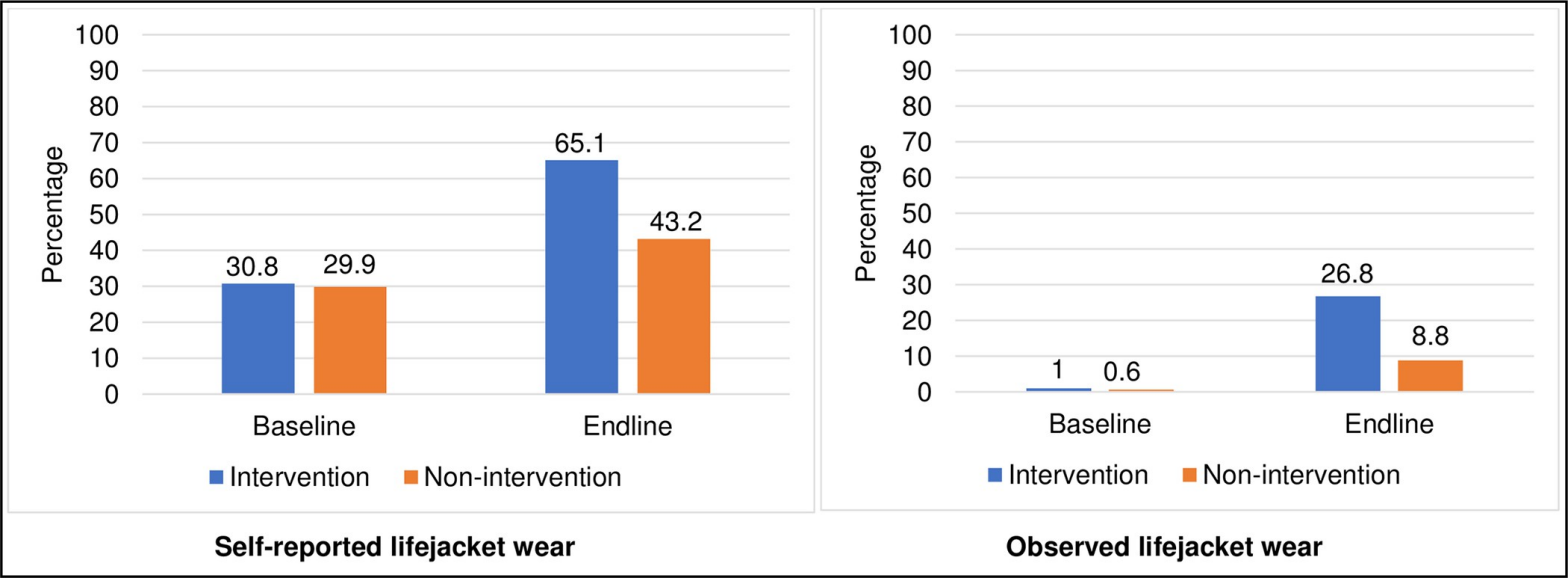
Effect of peer-led training on lifejacket wear among occupational boaters on Lake Albert.

Self-reported lifejacket wear increased markedly from 30.8% to 65.1% in the intervention arm than in the non-intervention arm which rose from 29.9% to 43.2% at endline. Observed wear rose from 1.0% to 26.8% in the intervention arm and from 0.6% to 8.8% in the non-intervention arm, Fig 3.

### Test of differences in proportions on lifejacket wear among occupational boaters on Lake Albert, Uganda

We conducted a test of differences in proportions of lifejacket wear to assess the effect of the peer-led training among occupational boaters. At baseline, self-reported lifejacket wear between the non-intervention and intervention arm was not significantly different (30.8%– 29.9% = 0.9%, p-value 0.743), indicating that both arms were comparable at baseline based on this variable, hence justifying the randomization. This was similar for observed lifejacket wear (1.0%– 0.6% = 0.4%, p-value 0.807). However, at endline, both self-reported and observed lifejacket wear were statistically different. Self-reported lifejacket wear was significantly higher in the intervention arm than in the non-intervention arm (65.1%– 43.2% = 21.9%, p-value <0.001). Observed lifejacket wear in the intervention arm was significantly different from that in the non-intervention arm (26.8%– 8.8% = 18, p-value <0.001), Table 3.

**Table 3:** Test of differences in proportions on lifejacket wear among occupational boaters on Lake Albert, Uganda.

Lifejacket wear	Baseline			Endline		
	Non-intervention N = 518	Intervention N = 629	P-value	Non-intervention N = 482	Intervention N = 487	P-value
**Self-reported**	155 (29.9%)	194 (30.8%)		208 (43.2%)	317 (65.1%)	
Difference	30.8%– 29.9% = 0.9%		0.743	65.1%– 43.2% = 21.9%		**<0.001**
**Observed**	Non-intervention N = 518	Intervention N = 629		Non-intervention N = 490	Intervention N = 474	
	3 (0.6%)	6 (1.0%)		43 (8.8%)	127 (26.8%)	
Difference	1.0%– 0.6% = 0.45		0.807	26.8%– 8.8% = 18%		**<0.001**

Estimated by linear regression

### Reach of the intervention

The results show that the intervention program spilled beyond the clusters that were assigned. About 16.8% (81/482) boaters in the non-intervention arm received peer training, indicating some level of contamination. In addition, 28.7% (140/487) of the boaters in the intervention arm did not receive the peer training, Table 4.

**Table 4:** Reach of intervention.

Variable	Intervention, n = 487, Yes (%)	Non-intervention, n = 482, Yes (%)
Heard about lifejacket campaigns	401 (82.3)	216 (44.8)
Attended a training session on lifejacket use	360 (73.9)	121 (25.1)
Received individual training from a peer	347 (71.3)	81 (16.8)

### Effect of contamination on the observed increase in self-reported lifejacket wear among occupational boaters on Lake Albert, Uganda

Because some individuals in the non-intervention arm received the peer-led training and some in the intervention arm did not receive (as shown in Table 3 above), the author performed further analysis to assess the effect of this spill-over in the observed increase in self-reported lifejacket wear. The Hosmer-Lemeshow (HL) model goodness of fit (GOF) test obtained a p-value of 0.092, indicating that the model fit the data reasonably well. The prevalence of self-reported lifejacket wear among the occupational boaters was almost two times higher among boaters who received training from their peers than those who did not (Adj. PR 1.78, 95% CI 1.38–2.30). It is interesting to note that this adjustment diminished the cluster effect, Table 5.

**Table 5:** Effect of contamination on the observed increase in self-reported lifejacket wear among occupational boaters on Lake Albert, Uganda.

Variable		Users/Interviewed,	Unadj. PR (95% CI)	Adj. PR (95% CI)	P-value
		n/N			
Age	Less than 20	8/25	1.0		
	20 to 29	231/411	1.41 (0.80–2.50)	1.32 (0.79–2.17)	0.284
	30 to 39	184/313	1.48 (0.81–2.71)	1.40 (0.83–2.37)	0.212
	40 to 49	68/142	1.31 (0.70–2.45)	1.21 (0.68–2.14)	0.513
	50 and above	34/78	1.29 (0.61–2.70)	1.15 (0.61–2.16)	0.669
Study arm	Non-intervention	208/482	1.0		
	Intervention	317/487	1.51 (1.01–2.22) *	1.09 (0.77–1.52)	0.629
Received training from peers at the landing site	No	207/541	1.0		
	Yes	318/428	1.84 (1.42–2.34) **	1.78 (1.38–2.30)	**<0.001**
Know how to swim	No	90/210	1.0		
	Yes	435/759	1.30 (1.08–1.54) *	1.20 (1.00–1.44)	0.052
Ever witnessed a person drowning	No	210/366	1.0		0.474
	Yes	315/603	0.91 (0.77–1.08)	0.94 (0.80–1.10)	0.295

*P-value <0.05

**P-value <0.001

## Discussion

This study implemented and evaluated the effectiveness of a peer-led training program aimed at improving lifejacket wear to prevent drowning among occupational boaters on Lake Albert, western Uganda. At endline, the results showed a significant increase in both self-reported and observed lifejacket wear. The test of differences in proportions of lifejacket wear showed that self-reported and observed wear significantly increased in the intervention arm. These differences were too big to have occurred by chance. Using the As Treated (AT) analysis demonstrated that the prevalence of self-reported lifejacket wear among the boaters who received the peer-led training was nearly two times higher than that among the boaters who did not receive the training (Adj. PR 1.78, 95% CI 1.38–2.30). It was interesting to note that the effect of the intervention arm was not significant. This means that being in either study arm was not important in this study. The results therefore demonstrate that the peer-led training is effective independent of the study arm, meaning that training implemented in the non-intervention group was effective in improving lifejacket wear. However, the main objective of this study was to test the effectiveness of the peer-led training on improving lifejacket wear.

Based on these results, contamination in this study was not necessarily a bad thing because the results show that the intervention can be effective even in the non-intervention arm. Whereas a systematic review of drowning prevention interventions argued that education alone is not effective [49], the author believes that this peer-led training augmented by peer pressure, increased lifejacket wear. In another systematic review of factors associated with lifejacket use, role modelling was a predictor of lifejacket wear among adolescents and indigenous communities [50]. Other studies have shown that training on lifejacket wear improves wear practices [18, 20, 51–54]. However, it should be noted that these studies largely focused on recreational boaters in high income settings while our study focused on occupational boaters in a low resource setting. Similar results have been noted elsewhere that peer training on lifejacket wear is effective [19, 51, 55, 56]. These results concur with the proposition that training of boaters can have a high population attributable fraction (PAF) in increasing lifejacket wear [57].

It is important to note that there was a significant increase in lifejacket wear even in the non-intervention arm. There are some postulates to this increase. To begin with, during the study period, the Police and Army Marine units intensified surveillance on the lake to fight the use of illegal fishing gear and rebel invasion into the country from the DRC. These operations may have had a positive non-intended effect on the use of safety equipment, including lifejacket wear as demonstrated to be effective elsewhere [21, 58]. In order to appear as law abiding boaters, they may have improved their safety practices including wearing lifejackets while on the lake. Furthermore, it is possible that the changes on weather conditions on the lake resulting in turbulent water waves could have forced the boaters to wear lifejackets more often. This study was however conducted at a season when the waves were waning and the turbulence subsidizing. A possible explanation for the contamination could be that given the transient nature of the boaters, even the people on the non-intervention arm may have received some training from their colleagues, while some boaters in the intervention arm may not have received the training. The boaters also may visit their friends and relatives who could be residents in the intervention sites and may receive the intervention. However, only boaters who were residents for at least three months in their respective landing sites were interviewed.

The results also show that the majority of the respondents reported to have received training on lifejacket wear from their peers at their respective landing sites. We believe that this peer-led training, which may have been augmented by positive peer pressure, increased lifejacket wear. These results therefore provide sufficient evidence to reject the null hypothesis that the peer-led training and routine marine community policing program has the same effect on lifejacket wear among the occupational boaters on Lake Albert, Uganda. A systematic review of drowning prevention interventions argued that education alone is not effective and there is need to integrate interventions [49]. However, our intervention incorporated both peer training (imparting skills on lifejacket donning procedures), education (sensitization) augmented by peer pressure. These are all important intervention functions under the COM-B and TDF model for behavior change [59, 60].

There was borderline significance in lifejacket wear among people who knew how to swim. This was not different from other studies in the same area and elsewhere that found that swimming expertise did not seem to have any effect on lifejacket wear [29, 50, 61, 62]. It would be expected that people who know how to swim also understand the risks associated with water and therefore would use lifejackets more often. This was not the case. Overestimation and over confidence alongside perceived low risk of drowning tagged on swimming expertise is a major risk factor for drowning [63, 64]. We also noted that having a lifejacket did not necessarily translate into wear, a similar result observed in an earlier study in the same community [29]. The country still faces a policy and regulatory deficit in implementing mandatory lifejacket wear because of lack of regulations to enforce the newly enacted In-Land Water Transport Act [24].

This study has some limitations. We relied on the self-reported lifejacket wear in the multiple regression model. We recognize that this may be subject to information bias, especially the expectation bias which might have made the participants report what they felt was acceptable to hear. However, we tried to validate this through covert observation of lifejacket wear as the boaters disembarked. The covert approach was chosen because it reduces the hawthorne effect whereby study participants may modify their behavior by putting on their lifejackets if they knew that they were being observed [37]. We were however not able to use this observed wear in the final model building because the observation was independent of self-reported wear and so the variables could not be matched with the interview respondents to infer associations. We are convinced by our results that there is a general improvement in lifejacket wear especially in the intervention arm as evidenced by both self-reported and observed results. Using the As Treated analysis meant that randomization has been lost. However, the main objective of this study was not to test the effectiveness of randomization but rather the peer-led training program.

While it is possible that police and army marines’ operations against illegal fishing may have had a non-intended impact on lifejacket wear, this should have affected both study arms equally. Our interviews started at 7:30 am and therefore possible that we may have missed out on the boaters who returned earlier than that time. In addition, a qualitative process evaluation leaning on critical realism would be helpful to explain how the intervention worked, for whom it worked and why. Lastly, due to the diverse cultural orientations among the different fishing communities in Uganda, this study cannot be generalized to all boaters in the country because it was conducted among boaters on only one lake. We however believe that our sample was powered enough to represent the occupational boaters in the districts around Lake Albert.

## Conclusion

This study demonstrates that regardless of the study arm, receiving peer-led training significantly improves lifejacket wear among occupational boaters on Lake Albert. Based on these results, there is sufficient evidence to reject the null hypothesis that peer-led training on lifejacket wear is not different from the routine marine community policing program among the occupational boaters on Lake Albert, Uganda. The government of Uganda through the respective ministries and the Landing Site Management Committees should embrace and scale up the peer-to-peer training on lifejacket wear to occupational drowning.

### What is already known on this topic

Behavioral interventions have been successful in improving lifejacket wear and consequently reduced the burden of drowning. However, these interventions have largely focused on leisure and recreational boaters in high-income countries. It is difficult to generalize these interventions to occupational boaters in rural low-income settings.

### What this study adds

This study demonstrates that peer led training improves lifejacket wear among occupational boaters in a rural low-resource setting. The peer pressure seems to enhance the effectiveness of the usual training and reminders to wear lifejackets to prevent drowning among the boaters involved in the occupations of fishing and transportation.

## Supporting information

S1 Checklist CONSORT 2010 checklist of information to include when reporting a randomised trial*. (DOC)

S2 Checklist *PLOS ONE* clinical studies checklist. (DOCX)

S1 File (DOCX)

S1 Data (CSV)

S2 Data (CSV)
