# Supplementary material for: Peer-led training improves lifejacket wear among occupational boaters: Evidence from a cluster randomized controlled trial on Lake Albert, Uganda
Source: PLoS One. 2023 Oct 20;18(10):e0292754. doi: 10.1371/journal.pone.0292754 (PMC10588873; doi:10.1371/journal.pone.0292754)
Supplement: S1 File — (DOCX) [file pone.0292754.s003.docx]

### **Effectiveness of a behavior-change intervention aimed to improve lifejacket wear among boaters involved in occupational boating activities on Lake Albert. A cluster randomized controlled trial.**

### **Introduction**

Drowning is a process of experiencing respiratory impairment from submersion/immersion in a liquid (1, 2). The outcomes are classified as death, morbidity and no morbidity (2). Drowning is a public health threat in every region of the world, and the most at risk populations are those with frequent exposure to any kind of water body (2, 3). Globally, drowning is the 3^rd^ leading cause of unintentional injury death; accounting for 7% of the burden attributed to injuries (2). Over 90% of the estimated 230,000 annual global drowning deaths occur in low-and middle-income countries (LMICs) (2, 4, 5), where the rates are 15-20 times higher than those in high income countries (HICs) (2). Moreover, these global estimates do not include drownings from water transportation and flood disasters, which are common in LMICs (6).

The World Health Organization (WHO) African region is among the most affected globally with a death rate at 8/100,000 population (6). Although some African countries like South Africa have registered significant strides on drowning prevention, the rate still remains high at 3/100,000 population (7), compared to some countries such as Canada and Luxemburg (8, 9). Occupations such as fishing and transportation on water are among the most at risk of drowning (6, 10). For example, the Lake Victoria Basin Commission estimates 4000–5000 drowning deaths each year; the majority of which occur among fishermen (11).

Death from drowning can occur at any of the 4 stages: 1- Cold shock; which may lead to hyperventilation, muscle spasms, increased heart rate and eventually cardiac arrest; 2- Swimming failure which usually proceeds complications initiated from stage one; 3- Hypothermia and 4- Post rescue collapse (12). Following these stages, unless there is immediate rescue and resuscitation, heart failure can occur within two minutes of total immersion (13). Although lifejackets prevent drowning, their greatest efficacy is only realized in stages 1 and 2 (12). Moreover, regardless of one’s swimming ability and water temperature, survival time when wearing a seaworthy lifejacket is over 7 times longer (14), ranging from 5-36 hours when water temperature is 15°C (12). The use of lifejackets has been greatly encouraged for people with frequent access to water (15).

### **1.2 Background**

Drowning death rates in lakeside fishing communities in sub-Saharan Africa are highest globally (16-18), with Uganda contributing the highest recorded rate of all; estimated at 502 per 100,000 population (16, 19). Fortunately, between 80-94% of lives can be saved when wearing a seaworthy lifejacket (10, 20, 21). As noted by the WHO, improving behavior on lifejacket use can be addressed through behavioral interventions using education, mandatory regulations and enforcement (22). However, although these interventions have been effective in increasing knowledge and use of lifejackets among recreational boaters in HICs (23-27), their effectiveness among boaters involved in occupational activities in rural low-income settings has not been measured.

Generally speaking, Uganda is gifted with many natural water resources. About 18% of the country’s land surface is covered by water (28) and over 38 districts, with a population of over fourteen million people (29) either bordering or surrounded by a water body (30). This resultantly points to inevitable frequent access to water, thereby increasing the risks of drowning. Indeed, there are many drowning incidents that have been reported in many media platforms (31, 32). The majority of these drownings are fatal. Although it is the role of government to ensure safety of lives on water, there is currently no law regarding the use of personal flotation devices (PFDs) such as lifejackets. Moreover, non-use of PFDs is one of the risk factors for drowning (19, 33, 34).

Lifejacket wear in Uganda is generally low. A recent countrywide survey found that 95% (n=1,007) of the people who drowned from a boat were not wearing a lifejacket, the majority of whom were fishermen and those involved in transportation of passengers and goods (35). The most commonly reported reasons were insufficient knowledge on the effectiveness of lifejackets alongside active distrust about the seaworthiness of the lifejackets available locally. There is a general belief that the lifejackets on the local market are ineffective in preventing drowning (19, 35). Moreover, it was observed that the majority of people incorrectly donned their lifejackets, a practice that may increase the risk of drowning.

A lifejacket is considered seaworthy if it meets the in-water performance and mechanical requirements of safety of life at sea (SOLAS) (36, 37) . It must also be used according to the prescribed user body-weight and in the right type of water body (38, 39). Although there are some known outlets that supply seaworthy lifejackets in Uganda, the majority of Ugandan boaters do not know how to select the right lifejacket for their body weight or one that is suitable for the type of water body (35). If a lifejacket is to perform as expected, it should be of the right fit and right type of water body (20, 40). Boaters need to be equipped with this vital knowledge in order to improve safety on water. However, there is a paucity of literature addressing this gap in Uganda. This study seeks to determine the effectiveness of a behavior-change intervention on improving lifejacket wear among boaters in Uganda?

### **5.3.8** **Study design**

Given that the intervention will be at community level, a cluster randomized controlled trial is considered most appropriate for testing the hypothesis of this study (41-43). The intact groups/clusters of individuals (boaters) rather than individuals themselves, will be randomized to either intervention or control arm (43). The landing sites, herein referred to as clusters, will be the randomization unit. This is because it is difficult to randomize individuals to a behavioral intervention, since they mix within the cluster. This study will be conducted with the undertones of an etic epistemological approach leaning a realist ontology to objectively measure the effect of the intervention.

### **5.3.9 SAMPLE SIZE**

All the functional and accessible landing sites will be included in the study. Using a sample size determination formula for comparing two proportions as expressed by Hayes and Bennett (44), the number of individuals per arm is determined as follows:

N_per group_: = $\frac{(Z_{\alpha/2}+Z_{\beta})^{2}\left[ P_{1}(1-P_{1}))+P_{0}(1-P_{0})) \right]}{\left( P_{1}-P_{0} \right)^{2}}$

Where:

N is the number of individuals per arm.

$Z_{\alpha/2}$ two-sided Z-value assumed alpha of 0.05, for Pearson’s Chi Square test at 95% confidence interval (1.96), i.e., the probability of rejecting the null when the null is true (probability of concluding that the intervention is better when in fact it is not).

$Z_{\beta}$ statistical power to detect a difference between the two proportions if such a difference exists, at 90% (1.28), i.e., the probability of detecting an effect of the intervention if that effect exists.

$P$_0_ = proportion of lifejacket wear in the non-intervention arm. This is got from the baseline survey that found self-reported lifejacket wear at 31.9% among the boaters on Lake Albert, Uganda (45). Therefore, the number of respondents will be 97 per arm. This will be inflated by a design effect of 5.1 obtained using the intracluster correlation coefficient (ICC) of 0.12 from the baseline survey (45) and a cluster size of 35.

$P$_1_ = proportion of lifejacket wear in the intervention arm. This study hypothesizes to increase lifejacket wear to 55% (0.55) after a six-months intervention. It is hoped that the clinical importance of this change is in terms of reduction of the risk of drowning. This change is clinically significant because the number of people using lifejackets will have increased and therefore reduced the risk of drowning. Following these assumptions, the final sample size was 495 boaters per arm, giving an overall total of 990 boaters.

### **5.4 Eligibility criteria**

This study will exclude clusters/landing sites that are known to have ongoing, or have had behavioral interventions on lifejacket use, except for the Marine Police routine sensitizations. It will also exclude clusters that have less than 55 boaters because this is the expected number per cluster, and clusters that have been flooded by the rising lake water levels. At individual level, the study will include boaters who are Ugandans and are aged 18 years and above or are emancipated minors where applicable. The boater should have lived and operated a fishing or transport boat at any landing site in Uganda for not less than one month at the time of the study. Boaters who transport passengers and merchandize to the DRC will be excluded in an attempt to reduce the possible confounding effect of compulsory lifejacket use in the DRC. Other factors which, in the opinion of the investigator, might interfere with the validity of the outcome will be considered as justification for exclusion or inclusion.

#### **5.5** **Randomization**

As illustrated on Figure 1, to reduce on covariate imbalance and increase comparability at baseline, clusters were randomized to either intervention of control arm using stratified permuted block randomization (42), with landing sites (clusters) as the randomization unit. The clusters were stratified by estimated population size, baseline prevalence of lifejacket wear and availability of shops that sell lifejackets locally (the clusters that had such shops were matched). The main aim of the stratified randomization is to ensure that the clusters are as comparable as possible at baseline. Because there are few clusters per arm (7), two strata will be formed (46). The main stratification variable was the baseline prevalence of lifejacket wear in the landing sites.

The risk of contamination (one of the major threats to this study) was reduced by a geographical buffer (at least one cluster which is not part of the study) between the intervention and control cluster. From each stratum, one cluster was randomly allocated to intervention arm and the other to control arm using stratified permuted block randomization with a 1:1 allocation ratio. The randomization was done by an independent statistician who is had no interest in the study who then generated the randomization sequence. The block sizes were masked/blinded to increase the randomness.

Eligible clusters at baseline

**Figure 1: Study schema for CRT**

Stratify

Don’t wear lifejackets

Wear lifejackets

Don’t wear lifejackets

Wear lifejackets

End line assessment

End line assessment

Intervention

Control

Randomize

***5.6 Administration of intervention***

The administration of the intervention will depend on the type of intervention identified from the formative studies and the insight workshops with relevant stakeholders. However, based on literature, there are potential three intervention functions of the COM-B model of the behavior change wheel (47). These functions are education (increase knowledge and understanding), training (impart skill) and role modeling (providing example for people to get inspired) (47). Education will be conducted by providing theoretical sessions through physical sensitizations conducted once every 1 month for six months.

In addition, posters written in the local dialects and SMS reminders as per the Boating Safety Communications & Education Strategy of the New South Wales (48) will be used as another mode of delivery/communication. The SMS reminders will be sent every 3 weeks. The frequency of the messages and sensitizations sessions has been selected to reduce on participant fatigue (too frequent messages). Secondly, these timings have been chosen with the assumption that they will provide a detectable dose-response relationship, as was done in a previous quasi experimental study among fishing communities in Lake Victoria, central Uganda (49). Phone contacts of every boater in the clusters will be obtained from the leadership of the Boat Owners’ Association, and so all the boaters in the intervention arm will receive the SMS messages.

The physical sensitization sessions will be accompanied by training through demonstrations on correct procedures of donning a lifejacket. Return demonstrations by the trainees will be conducted. Role modeling will be done using drowning survivors who used a lifejacket. The role models will be used as peer trainers and champions of lifejacket use. They will be provided with a standardized training package/manual that has the same messages on sensitizations by the study team. They will be ‘reminders’ of boaters to wear lifejackets every time they go to the lake. In case a legislative intervention is identified in objective 4, the local leadership will be trained and involved in the enforcement of lifejacket use. In order to maintain internal validity of the study, especially exposure identification bias, double blinding will be done. The RAs and the respondents will not know which cluster was allocated to the intervention or control arm.

In the control arm, the Uganda Police Marines will be encouraged to continue with their regular conduct of community policing through sensitizations on safety on water, including lifejacket use and other drowning prevention messages. These are standardized leaflet messages (Appendix J) designed by the Marine Police unit to be used for sensitization of communities on drowning prevention and other water safety issues.

At the time of consenting, the participants will be informed about the possibility of being in either the intervention or control arm. After randomization, allocation will be concealed from the participants so that they will not know which study arm they are in. Double blinding is preferred so that the RAs do not influence the outcome by treating respondents in the intervention group differently from those in the control group, thereby increasing objectivity by the RAs and reducing on risks of exposure identification bias, especially interviewer bias. Similarly, the respondents will not know whether they received the intervention or not since even the control arm will continue receiving the routine Marine Police messages. Contacts of survivors will be obtained from the previous survey. In addition, the local leadership of the landing sites and witnesses will be used to identify drowning survivors who may not be in the current list.

#### **5.7 Data analysis**

End line data will be imported to STATA 14 statistical software for analysis. Univariate, bivariate and multivariate analysis will be conducted using multilevel modeling (cluster level model and boater level model) with focus on the individual as the unit of analysis while accounting for clustering (50). The generalized estimating equation (GEE) will be used to account for within cluster variations. The goodness of fit of the model will be tested using the Hosmer Lemeshow test. The hypothesis will be tested at 5% alpha level. Lifejacket use will be used as a proxy for change of behavior as the main outcome. In addition, various aspects of knowledge and attitudes on lifejacket use will also be measured post intervention. Although there may be little observed use, lifejacket ownership will be used as a proxy to measure intention to use a lifejacket. The results will be reported in accordance with the consolidated standards for reporting trials (CONSORT statement) (51). Comparison between the baseline and end line will be done and the test of differences to show the effect in the intervention arm.

### **5.8 Quality control**

The study will utilize the Research Assistants (RAs) who are working in the current drowning prevention project where this study is nested.

#### **5.8.1 Training of RAs**

The RAs will undergo a 3-day training to introduce them to all aspects of data collection including objectives, protocol, and standard operating procedures. Sufficient information will be provided to ensure clear understanding of how to access the leadership of the landing sites and identify the study participants. All data collectors will be provided with a manual that has pictures of the different types of lifejackets for reference while in the field. Their academic qualification is minimum of bachelor’s degree in health and social sciences related fields. Selection of the RAs will ensure they are fluent in Alur and Runyooro, the languages most widely spoken in the study area. The RAs who will be involved in data collection will be blinded from the team that administers the intervention, with the overall supervision of the principal investigator (PI).

#### **5.8.2 Data safety**

The data will be examined daily to ensure completeness. Restrictions will be used in the programming of the questionnaire in the ODK to have skip patterns and prompts based on responses to prior questions. Data will then be exported to STATA 14.0 (Statacorp Texas; USA) for analysis. The data collection locations will be confirmed using global positioning system (GPS) to record the geographical coordinates. For quality assurance purposes, average duration of the interviews will be monitored and compared to averages determined during the pilot test. The electronic database will be backed up on cloud server daily and will be kept secure using a password which will only be known to the data manager and the PI. The cloud server will be hosted at Makerere University School of Public Health. Every RA will be provided with a power bank as a backup power source to ensure continuity of the data collection exercise.

#### **5.8.3 Pre-testing**

Prior to full scale data collection, the data collection tool will be pre-tested and any issues that may arise will be resolved. This pre-testing will occur in the field, during the training of RAs. The tool will be pre-tested among the boaters in one of the landing sites on Lake Victoria.

### **5.9 Ethical considerations**

Approval will be obtained from the HDREC of MakSPH. This study will also be registered with the Uganda National Council for Science and Technology (UNCST). The leadership of the landing sites (clusters) and leaders of Boat Owners’ Association will be approached to give permission for the study and to obtain phone contacts of all the boaters. Written informed consent (Appendix A) will be obtained from participants aged over 18 years prior to participation in the study. Children who are caretakers of families, and aged between 13-17 will be treated as emancipated minors and therefore will be required to consent. For the lifejacket tests that require entry in water, only participants who consent to get wet and know how to swim will be recruited. For this purpose, Marine Police officers will be recruited. As mentioned earlier, to avoid exposure identification bias (interviewer bias), double blinding will be done; the RAs and the study participants will not know which cluster received the intervention. However, prior to this, participants will be informed of a possibility of being in the intervention or control arm. The investigator declares no conflicts of interest in promoting use of lifejackets that have been found seaworthy. For every respondent whose lifejacket will be taken for testing, a new seaworthy lifejacket will be given as replacement. In case the identified intervention necessitates use of SMS messages, phone numbers of all the boaters will be obtained from the leadership of the landing site and boat owners association.

### **5.10 Plan for dissemination of results**

The findings will be disseminated in several local, national and international conferences through oral or poster presentations. All the study findings will be published in open access peer reviewed journals. Lastly, the full thesis will be submitted to Makerere University for the award of the degree of doctor of philosophy (PhD).

### **5.11 Limitations**

This study anticipates some limitations. First, Lake Albert is shared by Uganda and the DRC where lifejacket use is mandatory by law. This might affect the outcome since the populations mix. The investigator will attempt to address this limitation by ensuring that only Ugandans are recruited into the study, and will also be controlled for at analysis. There may be possibility of cross over between intervention and control arms. To address this limitation, a buffer zone will be created by ensuring geographical separation skipping at least one cluster between the intervention and control arm.

This study acknowledges that behavior change is gradual and so there may be delayed observation of the influence of the intervention during the period of this PhD study. However, the outcomes that change within the study period such as knowledge and attitudinal beliefs will be measured. Lastly, the observation of the standard operating procedures (SOPs) of Ministry of Health to prevent Covid19 may limit the number of participants at a given session.

**References**

1. van Beeck EF, Branche C, Szpilman D, Modell JH, Bierens JJ. A new definition of drowning: towards documentation and prevention of a global public health problem. Bulletin of the World Health Organization. 2005;83:853-6.

2. WHO. Drowning World Health Organization; 2018 [Available from: <https://www.who.int/en/news-room/fact-sheets/detail/drowning>.

3. Weiss J, Committee on Injury V, Prevention P. Technical report—Prevention of drowning. Pediatrics. 2010.

4. Lukaszyk C, Ivers RQ, Jagnoor J. Systematic review of drowning in India: assessment of burden and risk. Injury prevention. 2018;24(6):451-8.

5. Tyler MD, Richards DB, Reske-Nielsen C, Saghafi O, Morse EA, Carey R, et al. The epidemiology of drowning in low-and middle-income countries: a systematic review. BMC public health. 2017;17(1):413.

6. WHO. Global report on drowning: preventing a leading killer: World Health Organization; 2014.

7. Saunders CJ, Adriaanse R, Simons A, van Niekerk A. Fatal drowning in the Western Cape, South Africa: a 7-year retrospective, epidemiological study. Injury prevention. 2018:injuryprev-2018-042945.

8. Clemens T, Tamim H, Rotondi M, Macpherson AK. A population based study of drowning in Canada. BMC public health. 2016;16(1):559.

9. Drownings [Internet]. World Health Rankings 2017 [cited 5th May 2020]. Available from: <https://www.worldlifeexpectancy.com/cause-of-death/drownings/by-country/>.

10. Pitman SJ, Wright M, Hocken R. An analysis of lifejacket wear, environmental factors, and casualty activity on marine accident fatality rates. Safety science. 2019;111:234-42.

11. LVBC. Maritime Communications For Safety On Lake Victoria (MCSLV). Lake Victoria Basin Commission. <https://scholar.google.com/scholar?hl=en&as_sdt=0%2C5&q=Maritime+Communications+For+Safety+On+Lake+Victoria&btnG>=: Lake Victoria Basin Commission; 2017.

12. Sally Turner, Julie Wylde, Martin Langham, Suzy Sharpe, Jackson. K. MCA Lifejacket Wear–Behavioural Change. 2009.

13. Noble C, Sharpe N. Drowning: Its mechanism and treatment. Canadian Medical Association journal. 1963;89(9):402.

14. Marine Notice No. 39 of 2013. Notice to all Owners, Charterers, Masters, Skippers and Crew of Fishing and Commercial Vessels, (2013).

15. Cortés LM, Hargarten SW, Hennes HM. Recommendations for water safety and drowning prevention for travelers. Journal of travel medicine. 2006;13(1):21-34.

16. Miller L, Alele FO, Emeto TI, Franklin RC. Epidemiology, risk factors and measures for preventing drowning in Africa: a systematic review. Medicina. 2019;55(10):637.

17. Franklin RC, Peden AE, Hamilton EB, Bisignano C, Castle CD, Dingels ZV, et al. The burden of unintentional drowning: global, regional and national estimates of mortality from the Global Burden of Disease 2017 Study. Injury prevention. 2020.

18. WHO. Drowning: Key facts <https://www.who.int/en/news-room/fact-sheets/detail/drowning>: World Health Organization; 2021. Contract No.: 17th March 2020.

19. Kobusingye O, Tumwesigye NM, Magoola J, Atuyambe L, Olange O. Drowning among the lakeside fishing communities in Uganda: results of a community survey. International journal of injury control and safety promotion. 2016;24(3):363-70.

20. Viauroux C, Gungor A. An empirical analysis of life jacket effectiveness in recreational boating. Risk analysis. 2016;36(2):302-19.

21. Cummings P, Mueller B, Quan L. Association between wearing a personal floatation device and death by drowning among recreational boaters: a matched cohort analysis of United States Coast Guard data. Injury prevention. 2011;17(3):156-9.

22. WHO. Preventing drowning: an implementation guide. World Health Organization; 2017. Report No.: 9241511931.

23. Wallis BA, Watt K, Franklin RC, Taylor M, Nixon JW, Kimble RM. Interventions associated with drowning prevention in children and adolescents: systematic literature review. Injury prevention. 2015;21(3):195-204.

24. Weiss J. Prevention of drowning. American Academy of Pediatrics. 2010;126(1):e253-e62.

25. Solomon R, Giganti MJ, Weiner A, Akpinar-Elci M. Water safety education among primary school children in Grenada. International Journal of Injury Control and Safety Promotion. 2013;20(3):266-70.

26. Leavy JE, Crawford G, Leaversuch F, Nimmo L, McCausland K, Jancey J. A review of drowning prevention interventions for children and young people in high, low and middle income countries. Journal of community health. 2016;41(2):424-41.

27. Bugeja L, Cassell E, Brodie LR, Walter SJ. Effectiveness of the 2005 compulsory personal flotation device (PFD) wearing regulations in reducing drowning deaths among recreational boaters in Victoria, Australia. Injury Prevention. 2014;20(6):387-92.

28. UN_WATER. National Water Development Report: Uganda. United Nations 2006.

29. UBOS. National Population and Housing Census 2014, Main Report, Kampala, Uganda. Uganda Bureau of Statistics Uganda Bureau of Statistics 2016.

30. Nsubuga FN, Namutebi EN, Nsubuga-Ssenfuma M. Water resources of Uganda: An assessment and review. Journal of Water Resource and Protection. 2014;6(14):1297.

31. Mugerwa F. 30 feared dead in Lake Albert accident. Daily Monitor. 2019.

32. Byaruhanga C. From revelry to tragedy on Uganda's party boat BBC: BBC; 2018 [Available from: <https://www.bbc.com/news/world-africa-46432020>.

33. Twesigomwe M. Legal protection against marine accidents in Uganda: Makerere University; 2014.

34. Whitworth HS, Pando J, Hansen C, Howard N, Moshi A, Rocky O, et al. Drowning among fishing communities on the Tanzanian shore of lake Victoria: a mixed-methods study to examine incidence, risk factors and socioeconomic impact. BMJ open. 2019;9(12).

35. Kobusingye O, Tessa Clemens, Frederick Oporia, Milton Mutto, Anthony Mugeere, Merissa Yellman, et al. Understanding and Preventing Drowning in Uganda. Technical Report for Stakeholders <https://news.mak.ac.ug/2021/05/mak-researchers-design-national-drowning-prevention-strategy/>: Makerere University School of Public Health 2020.

36. IMO. International convention for the safety of life at sea (SOLAS). London 1 November 1974 UN Treaty Series vol 1184 p 278. 1974.

37. SOLAS I. International Convention for the Safety of Life at Sea, 1974, as amended. Consolidated Edition, IMO, London. 2014.

38. APS-ADVISOR. Understanding the Different Types of Sailing Life Jackets: APS Advisor; 2017 [cited 2020 09/March/2020]. Available from: <https://www.apsltd.com/aps-advisor/life-jacket-types/>.

39. USCG. HOW TO CHOOSE THE RIGHT LIFE JACKET. A Handy Guide from Your United States Coast Guard. In: Guard USC, editor. USA. <https://www.uscgboating.org/images/howtochoosetherightlifejacket_brochure.pdf>: United States Coast Guard; 2010.

40. Macintosh R, Pask E. The testing of life-jackets. British Journal of Industrial Medicine. 1957;14(3):168.

41. Moulton LH. Covariate-based constrained randomization of group-randomized trials. Clinical Trials. 2004;1(3):297-305.

42. Bennett S, Woods T, Liyanage WM, Smith DL. A simplified general method for cluster-sample surveys of health in developing countries. World health statistics quarterly 1991; 44 (3): 98-106. 1991.

43. Donner A, Klar N. Design and analysis of cluster randomization trials in health research. 2000.

44. Hayes R, Bennett S. Simple sample size calculation for cluster-randomized trials. International journal of epidemiology. 1999;28(2):319-26.

45. Oporia F, Nuwaha F, Kibira SP, Kobusingye O, Makumbi FE, Nakafeero M, et al. Lifejacket wear and the associated factors among boaters involved in occupational boating activities on Lake Albert, Uganda: a cross-sectional survey. Injury Prevention. 2022.

46. Hayes R, Moulton L. Cluster randomised trials. Taylor & Francis; 2009.

47. Michie S, Van Stralen MM, West R. The behaviour change wheel: a new method for characterising and designing behaviour change interventions. Implementation science. 2011;6(1):42.

48. Maritime Management Centre N. Boating Safety Communications and Education Strategy 2015-18. In: Centre MM, editor. <https://maritimemanagement.transport.nsw.gov.au/documents/boating-safety-coms-ed-strategy-15-18.pdf>: New South Wales Government 2015.

49. Kunle O, Tumwesigye NM, Magoola J, Atuyambe L, Kobusingye O. Preventing drowning among Lake Victoria basin fishing communities in Kampala, Buikwe, Mukono and Wakiso districts in Uganda. 2017.

50. Goldstein H, Browne W, Rasbash J. Multilevel modelling of medical data. Statistics in medicine. 2002;21(21):3291-315.

51. Begg C, Cho M, Eastwood S, Horton R, Moher D, Olkin I, et al. Improving the quality of reporting of randomized controlled trials: the CONSORT statement. Jama. 1996;276(8):637-9.
